# Transforaminal Endoscopic Discectomy Under General and Local Anesthesia: A Single-Center Study

**DOI:** 10.3389/fsurg.2022.873954

**Published:** 2022-04-19

**Authors:** Talgat Kerimbayev, Yergen Kenzhegulov, Zhandos Tuigynov, Viktor Aleinikov, Yermek Urunbayev, Yerbol Makhambetov, Andrew Pan, Nurzhan Abishev, Meirzhan Oshayev, Dinara Baiskhanova, Makar Solodovnikov, Serik Akshulakov

**Affiliations:** Department of Spinal Neurosurgery and Pathology of the Peripheral Nervous System, JSC “National Center for Neurosurgery”, Nur-Sultan, Kazakhstan

**Keywords:** transforaminal discectomy, local anesthesia, general anesthesia, lumbar spine, herniation

## Abstract

Percutaneous spinal endoscopy is used for the treatment of disorders of the lumbar spine, as it has several advantages over traditional surgical methods. The performance of percutaneous spinal endoscopy is not possible without applying anesthesia methods. Two types (local and general) of anesthesia are used for percutaneous spinal endoscopy. Both, local and general anesthesia approaches contribute to safety in surgical procedures. Although it is believed that the method of local anesthesia has more benefits over general anesthesia, such as lowering the risk of postoperative neurological complications in a patient, the literature on the topic is inconclusive. The study aims to perform a comparative analysis of the two anesthesia methods using a prospective case-control design. Patients were divided into two groups: those who received local anesthesia (LA) (20 patients), and those who underwent general anesthesia (GA) (20 patients). As a result of the study, 40% of the patients experienced moderate pain and 5% of the patients experienced excruciating pain intraoperatively in the LA group. Although Visual Analog Scale and Oswestry Disability Index scores improved more rapidly in LA group, at the 12-month check-up point there was no significant difference between cases and controls. Nevertheless, there were postoperative complications such as nerve root injury in 10% of the patients; nausea, vomiting, dizziness, drowsiness in 15% of the patients in the GA group, and an insignificant or no such complications in patients of the LA group. The present study demonstrates that LA contributes to more positive short-term outcomes for patients as it facilitates nerve root damage prevention, and has no postoperative side effects on patients' well being.

## Introduction

The methods of surgical treatment of herniated intervertebral disks of the lumbar spine are progressing and evolving each year to minimize postoperative complications and unintended consequences. Although the gold standard of the herniated disc surgical treatment is the open microdiscectomy, recently, numerous techniques have been developed to minimize the trauma of the surgical approach without reducing the radicalness of the surgical operation (1).

One of such techniques is percutaneous endoscopic spine endoscopy, which is a minimally invasive procedure that can be used to treat a variety of lumbar spine disorders. Percutaneous endoscopic discectomy has demonstrated similar effectiveness to be open discectomy in treating lumbar disc herniation (2, 3). It has several advantages over the traditional open discectomy, such as less damage to the paravertebral muscles, less intraoperative blood loss, shorter postoperative hospitalization period, and early postoperative patient recovery. Percutaneous endoscopic lumbar discectomy is now becoming a routine operation in spine surgery. In most cases, percutaneous endoscopic discectomy is performed using the transforaminal approach, which can theoretically be used at all lumbar levels of the spine (4–6). However, it is sometimes difficult to perform transforaminal endoscopic discectomy for L5/S1 disc herniation due to the anatomical limitations in the lumbosacral region, such as the high iliac crest, large transverse spur of the L5 vertebrae, large facet joint, and narrow foraminal space (7).

Both general anesthesia (GA) and local anesthesia (LA) are commonly used in various endoscopic surgeries, including the transforaminal approach in the percutaneous endoscopic lumbar discectomy (8, 9). LA is recommended by most surgeons for the transforaminal approach in the percutaneous endoscopic lumbar discectomy, as it results in early functional recovery of patients, lowers nerve damage risks, and less intraoperative volume of blood loss. In addition, it allows surgeons to avoid pulmonary complications associated with the GA (10). Also, LA helps to reduce such frequent side effects of general anesthesia as sore throat, nausea, and vomiting, as well as headache and dizziness (11).

The LA approach also can lead to complications that may result in nerve root damage, rupture of the dura mater, hematoma, and intracranial hypertension (12). The patients' satisfaction and the surgeon's ability to perform prolonged surgery are two major benefits of GA.

There are still many debates about the feasibility, safety, and effectiveness of LA and GA in patients undergoing transforaminal endoscopic lumbar spine surgery. However, modern methods of GA, such as multimodal neopioid analgesia and accelerated approach, have not often been compared to LA. Thus, the present study aims to determine the type of anesthesia that provides the best clinical outcomes for patients undergoing the transforaminal approach in the percutaneous endoscopic lumbar discectomy.

## Materials and Methods

### Study Population

The present research is a prospective case-control study. Forty patients diagnosed with lumbar disc herniation and treated using the transforaminal endoscopic discectomy between January 2020 and July 2020 at the Spinal Neurosurgery Department of the National Center for Neurosurgery, Nur-Sultan, Kazakhstan were enrolled in the study. Patients were divided into two groups. The first group underwent GA and the second group received LA.

Patients who were diagnosed with single-level lumbar disc herniation, confirmed through magnetic resonance imaging (MRI), with typical irradiating pain in the legs, and failure of conservative treatment methods at least 3 months before the hospitalization were included in the study.

Patients with interlaminar endoscopic discectomy, lumbar spine stenosis or other spinal pathology, a history of lumbar spondylodesis surgery, extreme lateral lumbar spinal hernia, and active local or systemic infection were excluded from the study.

### Clinical Outcomes

Length of hospital stay (sum of days before and after the procedure), operation length, and intensity of intraoperative pain and neurological complications were recorded.

#### Study Instruments

Patients were asked to fill out the Visual Analog Scale (VAS) before the procedure, 3, 6, and 12 months after the procedure to measure the pain perception in the leg and back. The Oswestry Disability Index (ODI) was used to measure the severity of the functional disability that patients experienced due to herniated disc. Patients were asked to fill out the questionnaire before the procedure, 3 months after, 6 months after, and 12 months after the procedure.

#### Anesthesia and Surgical Procedure

Patients of the LA group were administered 10 ml of 0.5% lidocaine and 0.25% ropivacaine at a dose of 1.3 mg/kg to prevent associated pain. The group GA patients received 2–3 mg/kg propofol and 1 mg/kg fentanyl to facilitate endotracheal intubation. Muscle relaxation during intubation 1 mg/kg suxamethonium chloride, 0.05–0.02 mg/kg pipecuronium bromide.

**Figure 1:**
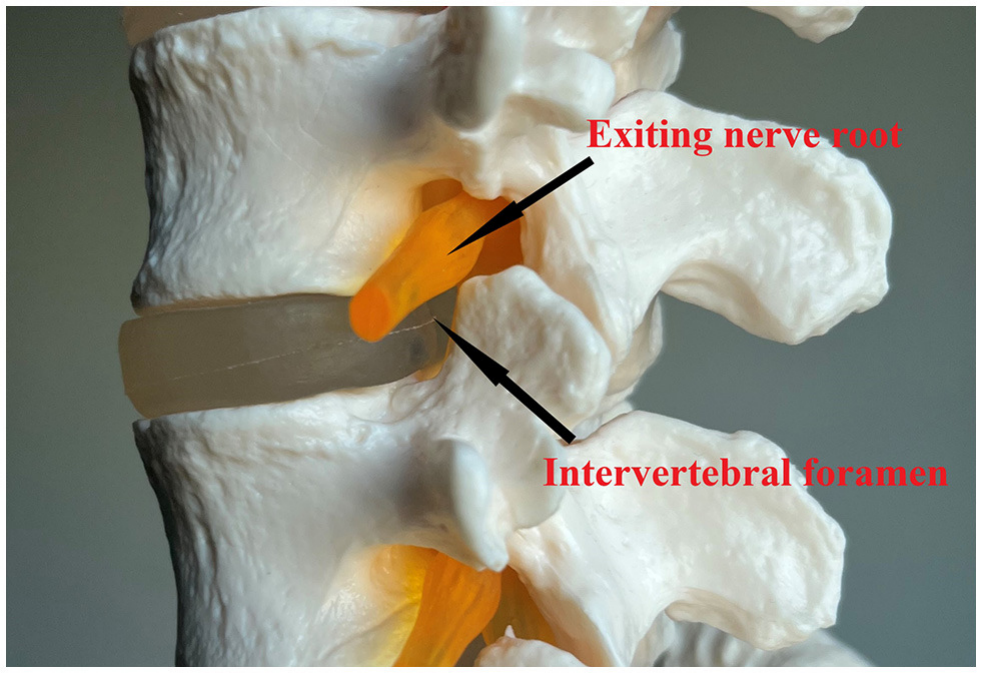
Anatomical location of the exiting nerve root, intervertebral foramen and safety triangle.

**Figure 2:**
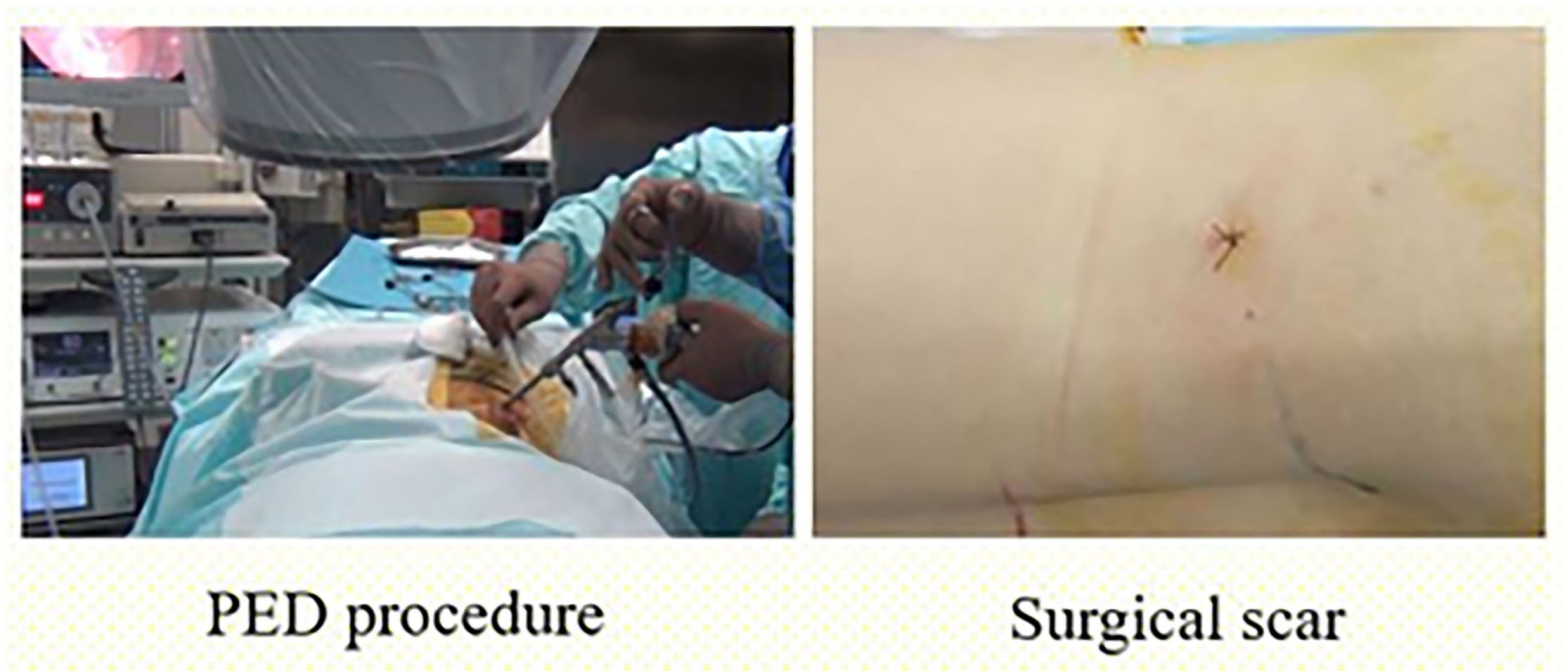
Operative scene in percutaneous transforaminal endoscopic discectomy and the surgical incisional scar.

**Figure 3:**
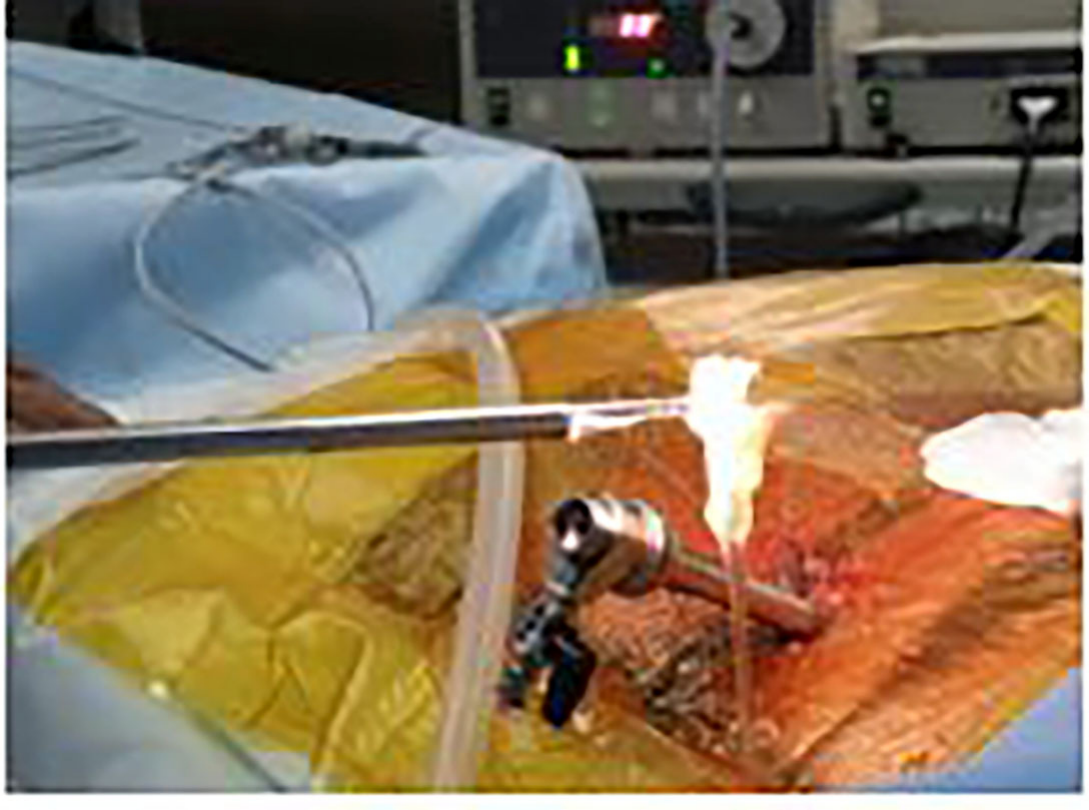
Removal of the herniated nucleus pulposus.

**Figure 4:**
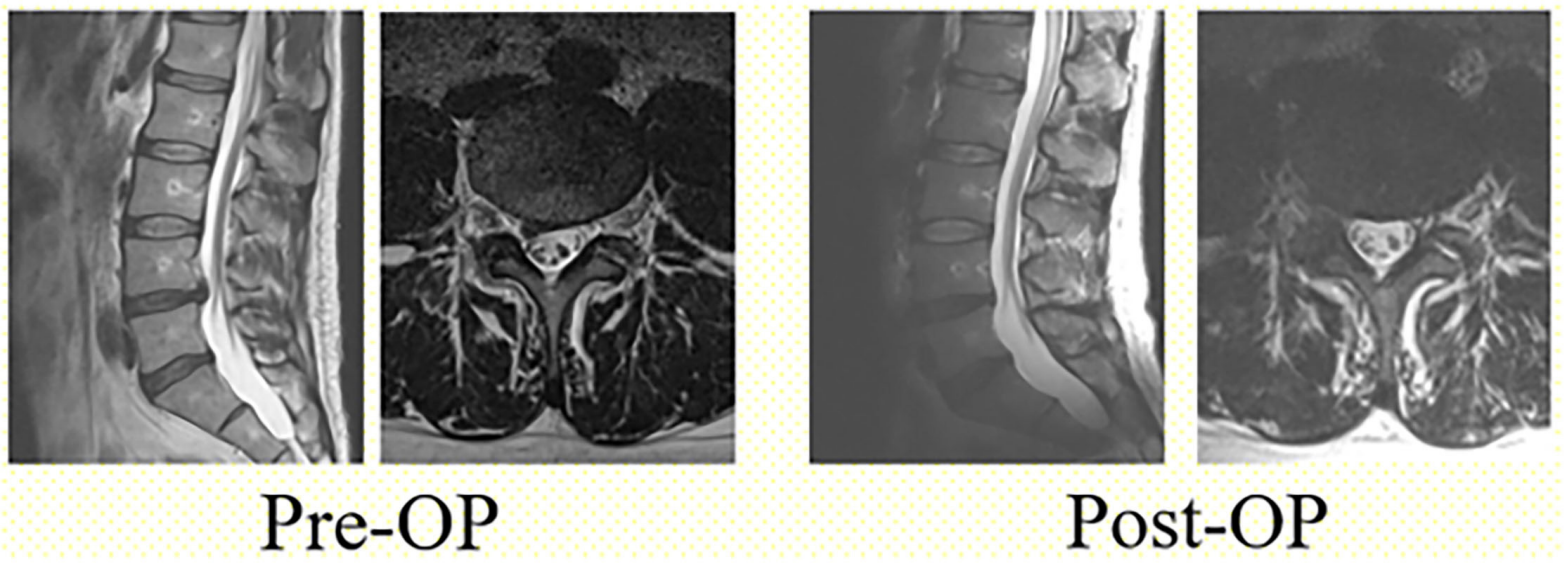
T2-weighted sagittal and axial views before and after surgery.

All surgical operations were performed with the endoscopic system (Richard Wolf Riwospine, Germany). The level of surgical intervention was determined using intraoperative fluoroscopy. The surgery was performed in the abdomen position on the spinal framework. The injection point of the cannula was determined preoperatively based on the CT and MRI images. The location of the cannula varied among patients depending on the anatomical and physiological characteristics and was ~8–12 cm from the midline. “The Walking Technique” was used to ensure safe access to the herniated glomerular nucleus through the safety triangle (Figure 1) Puncture needle was contacted with the caudal pedicle avoiding damage to the nerve root that exits from the cranial side of the intervertebral foramen. Then, using the walking technique, the needle was inserted into the intervertebral disc. A guide pin was inserted into the disc *via* the puncture needle, and the obturator and cannula were inserted sequentially through an 8-mm skin incision (Figure 2). After the cannula was inserted, the disc fragment at the base of the hernia was removed. Thereafter, using the inside-outside-down technique, the cannula was advanced toward the epidural space, the herniated mass was removed (Figure 3), and the pulsation of the dural tube was confirmed as an indicator of decompression (Figure 4). Patients were discharged when no signs of inflammation in the surgical wound were observed, and no pain syndromes were reported by patients.

The same surgical team, whose surgical experience exceeded 15 years in minimally invasive spine surgery, performed all the procedures.

#### Ethical Approval

Bioethics Committee of JSC National Center for Neurosurgery approved the study on 16 January 2020. The patient's written Informed Consent was obtained before the surgery.

### Statistical Analysis

The patients' answers on VAS and ODI surveys, as well as the demographic and clinical characteristics, were entered into Excel. The patient scores were calculated at pre-operational, 3, 6, and 12 months points. Together with demographic and clinical characteristics their pain and disability scores were entered and cleaned in Excel [Microsoft Office (Microsoft Corp., Redmond, Washington, USA)] and analyzed using STATA software, version 16.0 (Stata- Corp, College Station, Texas, USA). Descriptive statistics of the data consisted of percentages, means, standard deviations, and frequencies. Association of variables was tested with Student's *t*-test and Fischer or chi-square tests where appropriate. A threshold of 0.05 *p*-value was used for the determination of statistical significance.

## Results

The mean age of the participants was 46.9 ± 11.2 years old. No statistically significant difference was observed between cases and controls. The proportion of male patients was slightly higher (57.5%) than female patients, but no significant difference was presented between general and local anesthesia groups.

The hospital stay length of the general anesthesia group was significantly longer (5.1 ± 1.2 days), when compared to the local anesthesia group (3.5 ± 1.1 days). Operation time was also significantly shorter among the local anesthesia group (46.2 ± 9.3 min) when compared to the general anesthesia group (75.6 ± 5.5 min).

The VAS score in the back and leg was similar in both groups before the surgery. Three months after the procedure the pain decreased ~60% in the back and almost 75% in the leg with no significant difference between cases and controls. At 6 months point, those who were in the LA group reported an almost 78% decrease in back pain, while those in the GA group reported a 62% change. Change in leg pain perception followed a similar pattern with 87 vs. 75% decrease in local and general anesthesia groups respectively. There was no significant difference at 12 months point in the decrease of back pain between the groups (81% local, 82% general). However, the decrease in leg pain at 12 months was significantly higher in the local anesthesia group (99%), when compared to the general anesthesia group (86%) (Table 1).

**Table 1:** The comparison of variables between two experimental groups.

**Variable**	**General (mean ±SD)**	**Local (mean ±SD)**	***p*** **-value**	**Overall (mean ±SD)**
Age	47.6 ± 8.7	46.1 ± 13.5	0.68	46.9 ± 11.2
Sex				
Female	9 (52.94%)	8 (47.06%)	0.75	17 (42.5%)
Male	11 (47.83%)	12 (52.17%)		23 (57.5%)
Hospital stay (days)	5.1 ± 1.21	3.5 ± 1.1	<0.001	4.3 ± 1.4
Operation time (minutes)	75.6 ± 5.5	46.2 ± 9.3	<0.001	60.9 ± 16.7
VAS back before surgery	5.1 ± 0.9	5 ± 1.2	0.88	5.0 ± 1.1
VAS leg before surgery	8 ± 0.9	8 ± 1.1	1	8 ± 1.0
Change in VAS back 3 month (%)	57.5 ± 14.7	58.5 ± 16.9	0.85	58 ± 15.7
Change in VAS leg 3 month (%)	73.6 ± 9.1	75.1 ± 8.2	0.6	74.4 ± 8.6
Change in VAS back 6 month (%)	62.2 ± 17.4	77.9 ± 17.9	0.008	70 ± 19.2
Change in VAS leg 6 month (%)	74.8 ± 9.5	87.1 ± 7.6	<0.001	80.9 ± 10.6
Change in VAS back 12 month (%)	82.4 ± 14.2	80.8 ± 15.4	0.74	81.6 ± 14.7
Change in VAS leg 12 month (%)	85.8 ± 9.0	98.7 ± 4.1	<0.001	92.2 ± 9.5
ODI before surgery	50.0 ± 5.9	48 ± 8.2	0.39	49. ± 7.1
ODI 3 months	55.6 ± 6.1	61.8 ± 7.6	0.007	58.7 ± 7.5
ODI 6 months	59.4 ± 5.9	62.5 ± 7.3	0.14	61 ± 6.7
ODI 12 months	63.6 ± 4.3	63.8 ± 6.6	0.94	63.7 ± 5.5

**Figure 5:**
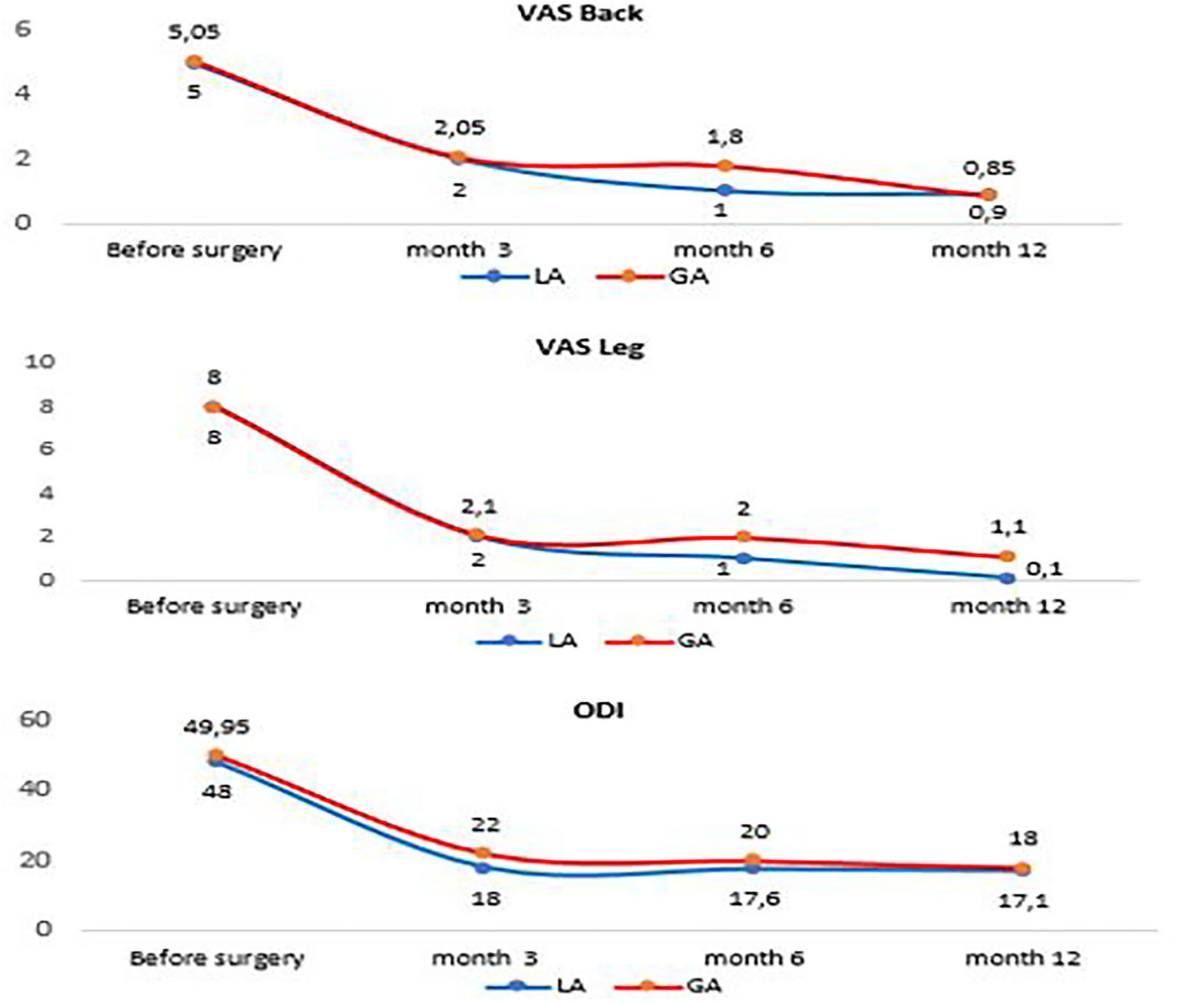
Comparison of ODI, VAS between the local group and the general group.

No significant difference was observed in pre-operational ODI between LA (48 ± 8.3) and GA (50 ± 5.9) groups. The decrease in ODI scores was significantly higher in the LA group (61.8 ± 7.6) when compared to the GA group (55.6 ± 6.1) 3 months after the surgery. Six and 12 months after the procedure the decrease in ODI was similar in both groups lowering by almost 64% (Figure 5).

In two patients in the GA group in a period of 5 months after the restorative treatment, patients' muscle strength had improved from grade 1 to grade 4. Patients in the LA group have reported a feeling of pain during surgery (40%) and excruciating pain (5%) during surgery. None of the patients in the GA group had such experiences. One patient (5%) in the LA group has experienced a nausea side effect. In the GA group 3 patients (15%) had experienced nausea, vomiting, dizziness, drowsiness, and 2 patients (10%) had sustained nerve root injury (Table 2).

**Table 2:** Comparison of adverse reactions, patient feeling between the local group and the general group.

**Adverse reactions and patient satisfaction**	**Local anesthesia 100% (***n*** = 20)**	**General anesthesia 100% (***n*** = 20)**
Nausea	5% (1)	15% (3)
Vomiting	0% (0)	15% (3)
Dizziness	0% (0)	15% (3)
Drowsiness	0% (0)	15% (3)
Nerve root injury	0% (0)	10% (2)
Pain during surgery	
Moderate pain	40% (8)	0
Excruciating pain	5% (1)	0

## Discussion

Several recent studies have shown LA as an effective, reliable, and successful alternative to GA in lumbar surgery (13, 14). The main drawback of GA is the sensory blockade that could lead to damage to the cauda equina nerve and nerve roots in patients, which is difficult to detect during surgery. For these reasons, most surgeons prefer LA to GA. However, LA has such disadvantages as surgical anxiety (15) and stress reactions caused by anesthesia, immunosuppression, and inflammatory processes (16). Wang and co-authors have found that continuous epidural anesthesia has more advantages than LA in improving the immune function of patients undergoing the percutaneous endoscopic lumbar discectomy for lumbar disc herniation. They also suggested that pain-free surgery would reduce adverse psychological effects, such as postoperative anxiety (17) and a recent study showed that patients prefer GA (18).

However, despite the disadvantages of LA mentioned above, this method still has more advantages over GA. For example, a neurosurgeon can control the patient's intraoperative pain during percutaneous endoscopic spine surgery. Keeping a patient awake plays a crucial role in spinal endoscopy to avoid nerve damage and allows the endpoint of surgery to be determined. In addition, LA does not require preoperative patient fasting and allows a physician to avoid some of the routine procedures required for GA, such as for example, tracheal intubation. This, in turn, contributes to a patient's rehabilitation immediately after the surgical procedure. In addition, the surgical procedure does not require drugs and devices associated with anesthesia and GA. For this reason, LA is less expensive than GA, which is an important factor that many surgeons should consider for their patients.

The present study aimed to examine the advantages and disadvantages of different anesthesia methods in transformational endoscopic discectomy, a less invasive surgical procedure that has shown to have minimal multifidus muscle atrophy (19, 20).

No postoperative infection was observed in both LA and GA groups. Despite the lack of complications observed in our LA group, a significantly larger proportion of patients have experienced discomfort in form of pain during the procedure. Currently, many surgeons are paying increasing attention to patient intraoperative psychology. Comfortable surgical experience is becoming increasingly important, as a successful surgical practice is associated with excellent postoperative clinical outcomes (21, 22).

Despite the sub-optimal patient experience during the surgery, the LA group had a shorter length of hospital stay and shorter surgery duration when compared to the GA group. This could be due to the preparation time for GA and recovery time after the tracheal intubation of a patient. Moreover, the LA group had more rapid improvement in VAS and ODI scores. Six months after surgery patients in the LA group had a significantly sharper decline in pain than in the GA group. However, the GA patients have caught up with LA patients by the 12 months mark. ODI score had a similar pattern with better recovery at 3- and 6-months points, but no difference in results between GA and LA groups at 12 months post-surgery. Only leg pain levels had remained significantly lower in the LA group 12 months after the procedure.

The GA group alone had postoperative complications such as nerve root damage in two patients, as well as nausea, vomiting, dizziness, and somnolence in three cases. Although the nerve root injury did not result directly from GA, the lack of communication with the patient during the surgery is one of the main factors that resulted in root injury. The frequency of dizziness, vomiting, and other symptoms has a direct relationship with anesthesia methods. Most drugs and anesthetics have a potential emetic effect, which had a higher association with general anesthesia in our study.

Considering the clinical outcomes of the patients in our study both GA and LA are effective methods for percutaneous transforaminal endoscopic surgery. Both groups have similarly recovered at 12 months follow-up point. However, the recovery in the LA group as well as the frequency of complications was significantly better than in the GA group. Physicians should take into account the somatic status of the patients, clinical outcomes, as well as their psychological comfort. Despite the discomfort that may occur during the surgery, the LA approach is a promising alternative to GA in percutaneous transforaminal endoscopic surgery.

## Data Availability Statement

The original contributions presented in the study are included in the article/supplementary materials, further inquiries can be directed to the corresponding author/s.

## Ethics Statement

The studies involving human participants were reviewed and approved by JSC National Center for Neurosurgery, Nur-Sultan, Kazakhstan. The patients/participants provided their written informed consent to participate in this study. Written informed consent was obtained from the individual(s) for the publication of any potentially identifiable images or data included in this article.

## Author Contributions

TK: conceptualization. YK: data collection. TK, VA, YU, YK, ZT, and AP: surgical procedures. MO and NA: postoperative observation of patients. YK and ZT: writing draft. DB, YM, and SA: review and editing of manuscript. All authors contributed to the article and approved the submitted version.

## Conflict of Interest

The authors declare that the research was conducted in the absence of any commercial or financial relationships that could be construed as a potential conflict of interest.

## Publisher's Note

All claims expressed in this article are solely those of the authors and do not necessarily represent those of their affiliated organizations, or those of the publisher, the editors and the reviewers. Any product that may be evaluated in this article, or claim that may be made by its manufacturer, is not guaranteed or endorsed by the publisher.
